# Cinnamic Acid Attenuates Ciprofloxacin-Induced Depression-like Behavior via Modulation of Neuroinflammation, Oxidative Stress, and Neurotransmitter Homeostasis in the Hippocampus–Prefrontal Cortex Axis

**DOI:** 10.3390/biology15141156

**Published:** 2026-07-15

**Authors:** Ares Alizade, Nur Akman

**Affiliations:** 1 Department of Pharmacology and Toxicology, Faculty of Medicine, Van Yüzüncü Yıl University, 65080 Van, Türkiye; 2 Department of Midwifery, Faculty of Health Sciences, Van Yüzüncü Yıl University, 65080 Van, Türkiye; nurakman@yyu.edu.tr

**Keywords:** ciprofloxacin, cinnamic acid, oxidative stress, neuroinflammation, cytokines

## Abstract

Ciprofloxacin (CPX) has been associated with neuropsychiatric adverse effects; however, its contribution to depression-like behaviors and the underlying neurobiological mechanisms remain insufficiently characterized. Previous studies have indicated that cinnamic acid (CA) may exert protective actions in the nervous system by modulating oxidative and inflammatory pathways. Nevertheless, its effectiveness in preventing CPX-induced neurobehavioral impairments remains largely unknown. The present study aimed to investigate whether CA attenuates CPX-induced depression-like behaviors through modulation of neuroinflammation and oxidative stress. This study included 60 male Swiss albino mice distributed among six experimental groups (*n* = 10). Animals were assigned to receive a vehicle, CPX administered at 80 mg/kg, CPX combined with CA at doses of 50, 100, or 200 mg/kg, or CPX together with FLX (20 mg/kg) throughout the 14-day treatment period. Behavioral alterations were assessed using social interaction, forced swimming, tail suspension, and sucrose preference paradigms. In addition, hippocampal and prefrontal cortex tissues were examined for oxidative stress markers (MDA, GSH, and CAT), pro-inflammatory cytokines (TNF-α, IL-1β, and IL-6), and neurotransmitters (serotonin, dopamine, and GABA) using ELISA-based assays. CPX administration induced significant depression-like behaviors, evidenced by reduced social interaction, increased immobility, and anhedonia (*p* < 0.05). These behavioral alterations were associated with increased lipid peroxidation, impaired antioxidant defense, elevated pro-inflammatory cytokine levels, and decreased monoaminergic and GABAergic neurotransmission in both HIP and PFC regions. CA treatment significantly improved CPX-induced behavioral and neurochemical alterations. Among the tested doses, the 100 mg/kg regimen produced the most consistent response, accompanied by reduced neuroinflammatory activity and restoration of neurotransmitter levels, particularly in the hippocampus. In conclusion, CA mitigated CPX-induced behavioral deficits and neurochemical alterations, an effect that may be attributed to its regulatory influence on oxidative stress, neuroinflammatory processes, and neurotransmitter balance within the HIP–PFC pathway. Collectively, the findings indicate that CA may have therapeutic relevance in reducing the neuropsychiatric consequences associated with CPX exposure.

## Simple Summary

Ciprofloxacin is a widely used antibiotic, but growing evidence suggests that it may also affect the central nervous system and contribute to behavioral changes. In this study, we investigated whether cinnamic acid, a naturally occurring phenolic compound found in many plants, could reduce depression-like symptoms induced by ciprofloxacin in mice. Behavioral tests showed that ciprofloxacin caused social withdrawal, increased immobility, and reduced sucrose preference, indicating depression-like behavior. These changes were accompanied by increased inflammation and oxidative stress, as well as reduced levels of important neurotransmitters in the hippocampus and prefrontal cortex. Treatment with cinnamic acid improved both behavioral and biochemical alterations, with a 100 mg/kg dose showing the most favorable overall response. The findings suggest that cinnamic acid may help protect against neurobehavioral disturbances associated with ciprofloxacin exposure by supporting antioxidant defenses, reducing inflammatory responses, and helping maintain neurotransmitter balance.

## 1. Introduction

Depression is a multifactorial mental disorder that influences emotional well-being, cognitive processes, and everyday functioning. Due to its high prevalence and persistent nature, it constitutes a major challenge for public health systems worldwide [1]. Epidemiological data indicate that depressive disorders are among the most important causes of disability and contribute substantially to the overall global disease burden [2].

The pathophysiology of depression arises from a complex interplay among neuroinflammation, oxidative stress, and dysregulation of neurotransmitter systems [1]. Higher levels of cytokines such as TNF-α, IL-1β, and IL-6 have been implicated in the activation of inflammatory pathways in the CNS, leading to alterations in synaptic function and neuronal integrity [3,4]. Concurrently, increased oxidative stress accompanied by insufficient antioxidant defense mechanisms exacerbates neuronal damage, further contributing to the development and progression of depression [3]. Alterations in serotonergic, dopaminergic, and GABAergic signaling have been implicated in many of the behavioral and cognitive abnormalities observed in depression [5].

The hippocampus (HIP) and prefrontal cortex (PFC) are regarded as key neural structures involved in the development and progression of depressive disorders [6]. The HIP is closely involved in the regulation of stress-related processes and contributes to the control of hypothalamic–pituitary–adrenal (HPA) axis activity [7]. Notably, stress-related conditions have been shown to disrupt synaptic plasticity between these regions and suppress mechanisms of long-term potentiation (LTP), thereby contributing to impaired neuronal communication and functional deficits [8]. Consistent with these findings, structural neuroimaging studies in individuals diagnosed with depression have demonstrated significant reductions in the volumes of both the PFC and HIP [9]. These observations are further supported by postmortem analyses revealing neuronal loss and decreased glial cell density in the same regions [9]. Such structural alterations have been linked to oxidative stress, neuroinflammatory activity, and disruptions in neurotrophic support mechanisms. Collectively, these pathological processes are considered to underlie the neurobiological basis of depression [10].

In recent years, accumulating evidence has suggested that ciprofloxacin (CPX), a widely prescribed fluoroquinolone antibiotic, may exert various adverse effects on the central nervous system (CNS) [11,12]. Experimental studies have demonstrated that CPX disrupts neurotransmitter homeostasis and impairs antioxidant defense mechanisms, [1,11,13] while simultaneously promoting the production of pro-inflammatory cytokines [1,11,12]. Collectively, these alterations suggest that CPX may contribute to the development of depression-like behaviors through mechanisms involving oxidative stress, neuroinflammation, and neurotransmitter dysregulation [11,12,13].

Fluoxetine (FLX), an antidepressant belonging to the selective serotonin reuptake inhibitor (SSRI) class, frequently employed in experimental models of depression, is a well-established reference drug with clearly defined antidepressant efficacy [14]. In addition to modulating serotonergic neurotransmission, FLX has been shown to suppress neuroinflammatory responses and enhance synaptic connectivity between the HIP and PFC [8]. Nevertheless, prolonged use has been associated with mitochondrial dysfunction and various adverse effects, underscoring the ongoing need for safer and more effective therapeutic alternatives [15].

Cinnamic acid (CA), a phenolic constituent identified in various plant species, has been investigated in different experimental settings because of its biological activities [16]. Available evidence indicates that CA can influence oxidative balance, inflammatory signaling, and neuronal integrity [17,18]. However, data regarding its effects in CPX-induced experimental depression remain limited.

Accordingly, the current study explored the effects of CA in a CPX-induced experimental depression model by examining behavioral changes, oxidative stress-related parameters, and neurotransmitter levels (serotonin, dopamine, and GABA) in the hippocampus and prefrontal cortex. We proposed that CA might improve CPX-induced behavioral abnormalities through modulation of oxidative and inflammatory pathways together with normalization of neurotransmitter signaling in the HIP and PFC. The findings obtained from this study may provide further insight into the biological processes involved in depression and offer additional evidence regarding the therapeutic potential of naturally derived compounds.

## 2. Materials and Methods

### 2.1. Chemicals

Ciprofloxacin hydrochloride (PHR1044, Sigma-Aldrich, St. Louis, MO, USA), fluoxetine (FLX, F132, Sigma-Aldrich, St. Louis, MO, USA), cinnamic acid (Cat. No: BD8127-10g, BLDpharm, Shanghai, China), ketamine hydrochloride (Ketaset^®^, Fort Dodge Animal Health, Fort Dodge, IA, USA) and xylazine hydrochloride (Rompun^®^ 2%, Bayer, Leverkusen, Germany) were employed for anesthesia procedures. Unless otherwise stated, all chemicals and reagents used throughout the study were of analytical grade.

### 2.2. Preparation of Chemicals

Cinnamic acid (CA) was prepared as a suspension in 0.5% carboxymethyl cellulose (CMC). Prior to administration, the mixture was thoroughly homogenized and delivered by oral gavage once daily during the experimental period [19]. Ciprofloxacin hydrochloride and fluoxetine hydrochloride were prepared in distilled water and administered via the oral route [11]. The CA suspension was administered orally by gavage once daily throughout the experimental period [19]. All treatments were administered orally by gavage once daily. In groups receiving combined treatments, CPX and CA or FLX were administered sequentially during the same dosing session.

### 2.3. Experimental Animals

The experimental study was conducted using 60 male Swiss albino mice aged 8–10 weeks, with body weights ranging from 25 to 35 g. Animals were supplied by the Experimental Medicine Research Center of Van Yüzüncü Yıl University (YÜDAM, Van, Türkiye).

Animals were maintained under controlled environmental conditions, including a temperature of 22 ± 2 °C, relative humidity of 50–60%, and a 12 h light/dark cycle. Standard pellet diet and water were available without restriction throughout the study. Only male mice were used in the present study to minimize potential variability associated with the estrous cycle and hormonal fluctuations, which may influence behavioral, inflammatory, oxidative stress, and neurotransmitter-related parameters.

All animal-related procedures complied with established ethical principles and animal care regulations. Ethical approval for the study was granted by the Van Yüzüncü Yıl University Animal Experiments Local Ethics Committee (Approval No: 2025/03-04; 27 March 2025).

### 2.4. Experimental Design and Grouping

Sixty mice were assigned to six experimental groups, with ten animals included in each group, and the study was conducted for 15 consecutive days. The treatment groups were defined as follows:

Group 1 (Control/Vehicle): Distilled water + 0.5% CMC.

Group 2 (CPX): Ciprofloxacin (80 mg/kg/day).

Group 3 (CPX + CA50): Ciprofloxacin (80 mg/kg/day) + cinnamic acid (50 mg/kg/day).

Group 4 (CPX + CA100): Ciprofloxacin (80 mg/kg/day) + cinnamic acid (100 mg/kg/day).

Group 5 (CPX + CA200): Ciprofloxacin (80 mg/kg/day) + cinnamic acid (200 mg/kg/day).

Group 6 (CPX + FLX, Positive control): Ciprofloxacin (80 mg/kg/day) + fluoxetine (20 mg/kg/day).

**Figure 1:**
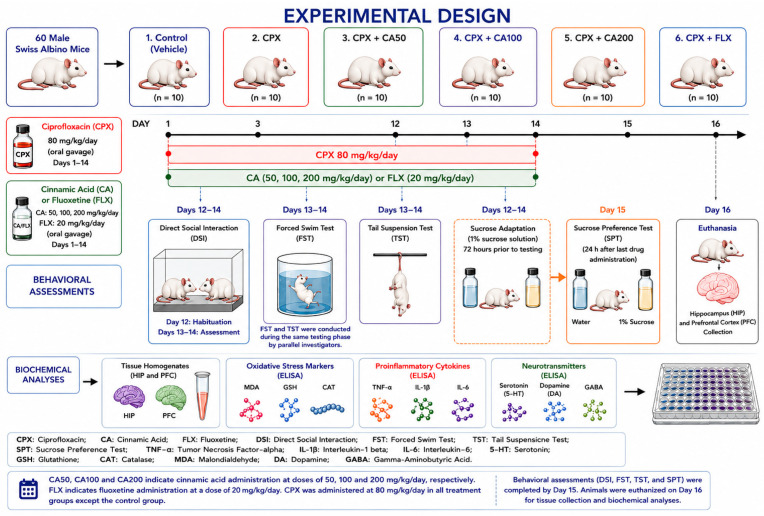
Experimental design of the study. Different colored frames indicate the experimental treatment groups, whereas arrows indicate the experi-mental timeline and sequence of procedures.

The selected doses of ciprofloxacin were based on previously established experimental protocols [20,21], while the doses of cinnamic acid and fluoxetine were chosen according to studies demonstrating their pharmacological efficacy [22]. The overall experimental design is illustrated in Figure 1.

### 2.5. Behavioral Assessments

Behavioral tests were performed during the final phase of the experimental protocol in the following sequence: the direct social interaction test (DSI; days 12–14), the forced swim test (FST) and tail suspension test (TST) (TST and FST; days 13–14), followed by the sucrose preference test (SPT) on day 15, 24 h after the final drug administration. DSI, FST, and TST assessments were conducted at the same time of day following daily treatment administration to minimize potential circadian and procedural variability. Behavioral assessments were conducted by two trained researchers working in parallel according to a predefined testing schedule. All experimental groups were evaluated under the same testing sequence and environmental conditions to minimize procedural variability and potential bias.

### 2.6. Direct Social Interaction Test

The test was conducted in an open-top Plexiglas arena measuring 40 × 40 cm. Each experimental mouse was paired with an unfamiliar conspecific mouse of the same strain and sex and similar age and body weight that had not previously been housed with or exposed to the test animal. The two mice were placed in opposite corners of the apparatus, and their interactions were observed for 7 min. During this period, the frequencies of social behaviors, including sniffing and grooming, were recorded [23].

The test was performed over three consecutive days. The first day was considered a habituation session to allow animals to acclimate to the environment, whereas behavioral parameters were recorded on the final two days and used for the assessment of social interaction. Behavioral data obtained on the two assessment days were analyzed and presented separately to confirm the stability and reproducibility of treatment effects across repeated testing sessions.

### 2.7. Forced Swim Test

The test mice were placed individually in a water-filled cylindrical container (40 cm in height and 18 cm in diameter) maintained at 24–25 °C. Behavioral activity was monitored for 6 min, and immobility time was evaluated during the final 4 min following an initial adaptation period.

Immobility was characterized by the absence of active swimming behavior, except for movements necessary to maintain flotation. After the assessment period, animals were taken out of the water, dried, and returned to their housing cages. Each mouse was evaluated once, and the test was performed according to previously published methods [24,25].

### 2.8. Tail Suspension Test

During the test, mice were fixed to a suspension apparatus by adhesive tape applied near the distal end of the tail. The apparatus was positioned 58 cm above ground level, and behavioral responses were recorded over a 5 min testing session.

Immobility was characterized by the lack of active body movements, apart from those related to respiration. Longer immobility durations were regarded as indicative of depression-like behavior, whereas shorter immobility periods were interpreted as evidence of an antidepressant-like response. The test was conducted according to previously published procedures [26,27].

### 2.9. Sucrose Preference Test (SPT)

Anhedonia-like behavior was evaluated using the SPT. The assessment was performed 24 h following the final treatment and was conducted as previously reported in the literature [28].

Animals were habituated to a 1% (*w*/*v*) sucrose solution for 72 h prior to testing. During the habituation phase, mice were provided with two bottles filled with 1% sucrose solution. Following habituation, one of the bottles was substituted with tap water, while the second bottle continued to contain the sucrose solution. Before testing, animals underwent a 24 h period of food and water deprivation and were subsequently housed individually. Each mouse was then given unrestricted access to two pre-weighed bottles containing either 1% sucrose solution or tap water. At the end of the test period, the volumes consumed from each bottle were recorded.

Sucrose preference was determined according to the following equation:

Sucrose preference (%) = [sucrose consumption/(Sucrose consumption + Water consumption)] × 100.

This test is widely accepted as a reliable method for assessing anhedonia-like behavior in experimental models of depression [28].

### 2.10. Hippocampus and Prefrontal Cortex Analyses

Following completion of the behavioral assessments, animals were anesthetized with ketamine (70 mg/kg) and xylazine (10 mg/kg) and subsequently euthanized by exsanguination. Brain tissues were immediately collected, and the HIP and PFC were isolated under cold conditions. Samples were processed in 0.1 M phosphate buffer (pH 7.4) to prepare 10% (*w*/*v*) tissue homogenates. After centrifugation at 4000 rpm for 10 min at 4 °C, the supernatant fractions were separated and used for biochemical analyses. Concentrations of neurotransmitters (serotonin, dopamine, and GABA), inflammatory cytokines (IL-1β, IL-6, and TNF-α), and oxidative stress-related markers (MDA, GSH, and CAT) were determined using commercially available ELISA kits in accordance with the manufacturers’ protocols. Serotonin, MDA, GSH, and CAT assay kits were supplied by BT LAB (Shanghai, China; Cat. Nos: E0866Ra, E0156Ra, EA0113Ra, and E0869Ra, respectively). Dopamine, GABA, TNF-α, IL-1β, and IL-6 kits were obtained from SunRed Biological Technology (Shanghai, China; Cat. Nos: DZE201020668, 201-02-0361, DZE201110765, DZE201110120, and DZE20102050, respectively). Optical density measurements were recorded at 450 nm using a Multiskan SKY Microplate Spectrophotometer (UV/VIS UV/VIS, Thermo Fisher Scientific, Vantaa, Finland) [29].

### 2.11. Statistical Analysis

Sample size was calculated to provide at least 80% statistical power while maintaining a type I error probability of 0.05. Data distribution was evaluated using the Shapiro–Wilk test together with skewness and kurtosis measures. Since the assumption of normality was not met, non-parametric analyses were employed. Data are summarized using measures of central tendency and dispersion, including mean, standard deviation, median, and range. Intergroup differences were analyzed by the Kruskal–Wallis test, followed by Bonferroni-adjusted post hoc comparisons where required. Statistical inferences were based on a significance threshold of 0.05. All computations were performed with IBM SPSS Statistics version 26.0 and GraphPad Prism version 8.0.

## 3. Results

### 3.1. Behavioral Outcomes

#### 3.1.1. Direct Social Interaction Test

**Figure 2:**
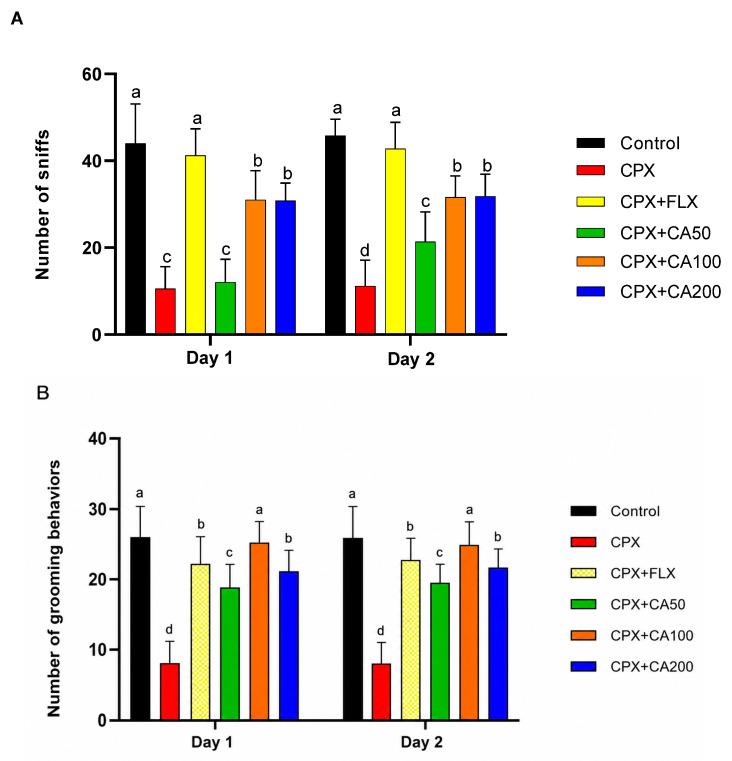
Evaluation of social interaction behaviors in the CPX-induced depression model. (**A**) Sniffing behavior. (**B**) Grooming behavior. Lowercase letters (a–d) above the bars represent the results of the multiple comparison analysis. Groups with different lowercase letters differ significantly (*p* < 0.05), whereas groups sharing at least one common lowercase letter are not significantly different according to the Kruskal–Wallis test followed by Bonferroni-adjusted post hoc comparisons.

Social interaction behaviors were evaluated based on sniffing and grooming parameters (Figure 2A,B). The CPX-treated group showed a significant reduction in both behaviors compared with the control group (*p* < 0.05). In the positive control group (CPX + FLX), sniffing and grooming behaviors significantly increased compared with the CPX group (*p* < 0.05). Similarly, CA treatment at doses of 100 and 200 mg/kg significantly increased both sniffing and grooming behaviors relative to the CPX group (*p* < 0.05). In contrast, the 50 mg/kg CA group exhibited a significant increase only in grooming behavior (*p* < 0.05), whereas sniffing behavior remained significantly lower than that of the positive control group (*p* < 0.05).

#### 3.1.2. Forced Swim Test (FST)

**Figure 3:**
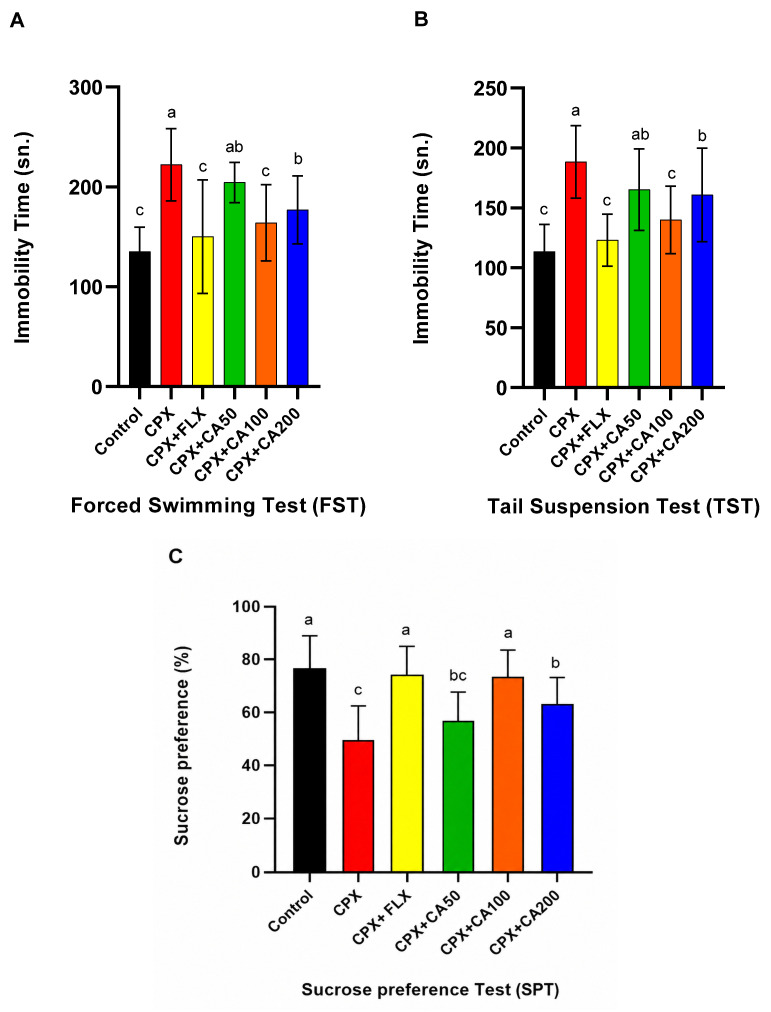
Behavioral outcomes in the CPX-induced depression model. (**A**) Forced swim test (FST) immobility time. (**B**) Tail suspension test (TST) immobility time. (**C**) Sucrose preference test (SPT) results. Data are presented as mean ± SD (*n* = 10). Lowercase letters (a–c) above the bars represent the results of the multiple comparison analysis. Groups with different lowercase letters differ significantly (*p* < 0.05), whereas groups sharing at least one common lowercase letter are not significantly different according to the Kruskal–Wallis test followed by Bonferroni-adjusted post hoc comparisons.

The outcomes of the FST are illustrated in Figure 3A. The CPX-treated group exhibited a significant increase in immobility time compared with the control group (*p* < 0.05). In the CPX + FLX group, immobility time was significantly reduced relative to the CPX group and approached control levels (*p* < 0.05). Similarly, the CPX + CA100 group showed a significant reduction in immobility time compared with the CPX group (*p* < 0.05). In contrast, no significant differences were observed in the CPX + CA200 and CPX + CA50 groups relative to the CPX group (*p* > 0.05). The 100 mg/kg CA treatment normalized immobility behavior, yielding immobility times that did not differ significantly from those observed in the control or CPX + FLX groups (*p* > 0.05).

#### 3.1.3. Tail Suspension Test (TST)

Animals in the CPX group exhibited longer immobility durations than those in the control group (*p* < 0.05). In contrast, the CPX + FLX group showed a significant reduction in immobility time relative to the CPX group (*p* < 0.05), with values comparable to the control group (*p* > 0.05). Among the CA-treated groups, a statistically significant decrease in immobility time was observed only in the CPX + CA100 group (*p* < 0.05), whereas no significant changes were detected at the other doses (*p* > 0.05) (Figure 3B).

#### 3.1.4. Sucrose Preference Test (SPT)

The CPX-treated group showed a significant reduction in sucrose consumption compared with the control group (*p* < 0.05). In contrast, sucrose preference was significantly increased in the CPX + FLX group relative to the CPX group (*p* < 0.05). Among the CA-treated groups, the CPX + CA100 group exhibited a significant increase in sucrose preference compared with the CPX group (*p* < 0.05), reaching levels comparable to the positive control group (*p* > 0.05). Although the CPX + CA50 and CPX + CA200 groups showed higher sucrose preference values than the CPX group, these differences did not reach statistical significance (*p* > 0.05) (Figure 3C).

### 3.2. Biochemical Findings

#### 3.2.1. Antioxidant System Markers

**Figure 4:**
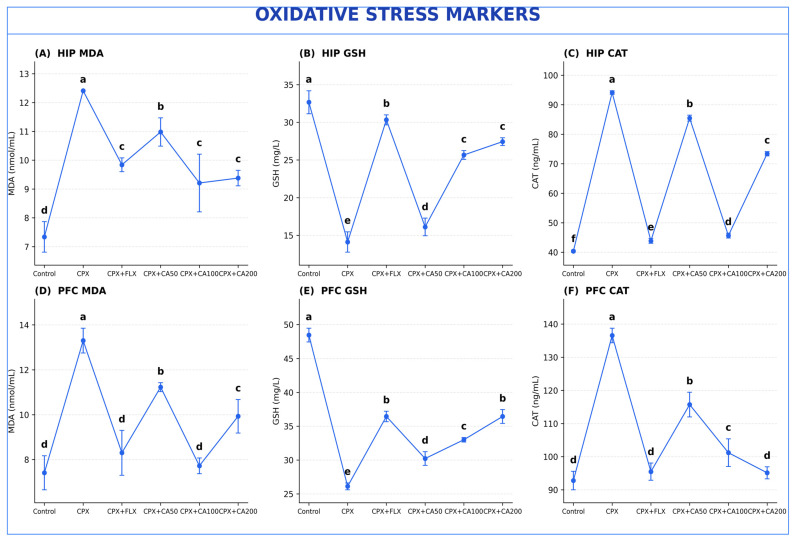
Effects of cinnamic acid (CA) on oxidative stress markers in the hippocampus (HIP) and prefrontal cortex (PFC) of mice exposed to ciprofloxacin (CPX). (**A**) HIP MDA, (**B**) HIP GSH, (**C**) HIP CAT, (**D**) PFC MDA, (**E**) PFC GSH, and (**F**) PFC CAT levels. Data are presented as mean ± SD (*n* = 10). Lowercase letters (a–f) above the bars represent the results of the multiple comparison analysis. Groups with different lowercase letters differ significantly (*p* < 0.05), whereas groups sharing at least one common lowercase letter are not significantly different according to the Kruskal–Wallis test followed by Bonferroni-adjusted post hoc comparisons.

Oxidative stress parameters in the HIP and PFC revealed that CPX administration induced marked biochemical alterations in both regions. Mice receiving CPX showed significantly higher MDA and CAT levels together with lower GSH concentrations than those observed in the control group (*p* < 0.05) (Table 1, Figure 4).

Among mice receiving FLX together with CPX, MDA concentrations were lower, whereas GSH levels were higher in both the HIP and PFC compared with animals exposed to CPX alone (*p* < 0.05). CAT activity also showed a reduction relative to the CPX group (*p* < 0.05). In contrast, treatment with CPX + CA50 resulted in higher MDA and CAT values and lower GSH concentrations than those observed in the CPX + FLX group (*p* < 0.05).

Animals administered CPX together with 100 mg/kg CA exhibited MDA values similar to those recorded in the positive control group (*p* > 0.05) and lower than those measured in CPX-treated animals (*p* < 0.05). However, CAT activity remained elevated and GSH concentrations remained reduced relative to the positive control group (*p* < 0.05).

The CPX + CA200 treatment group displayed hippocampal MDA concentrations that approached those of the positive control animals (*p* > 0.05). Nevertheless, GSH levels remained lower and CAT activity remained higher than positive control values (*p* < 0.05). Within the PFC, MDA concentrations exceeded those observed in the positive control group (*p* < 0.05), whereas no significant differences were detected for GSH or CAT levels (*p* > 0.05).

#### 3.2.2. Pro-Inflammatory Cytokine Levels

**Figure 5:**
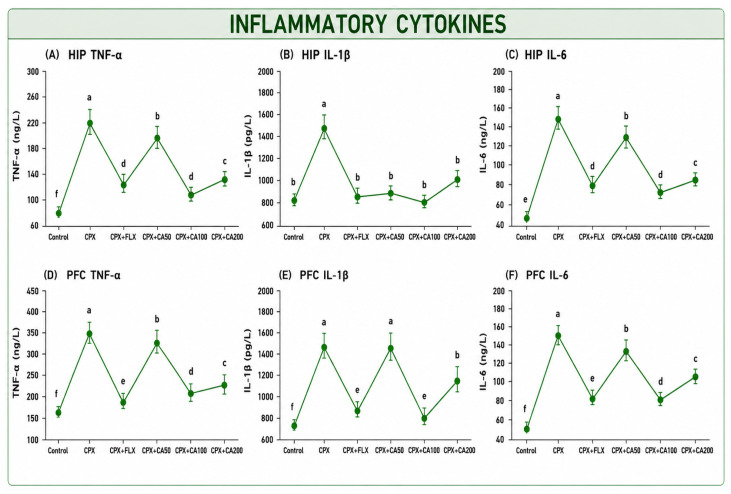
Effects of cinnamic acid (CA) on pro-inflammatory cytokine levels in the hippocampus (HIP) and prefrontal cortex (PFC) of mice exposed to ciprofloxacin (CPX). (**A**) HIP TNF-α, (**B**) HIP IL-1β, (**C**) HIP IL-6, (**D**) PFC TNF-α, (**E**) PFC IL-1β, and (**F**) PFC IL-6 levels. Data are expressed as mean ± SD (*n* = 10). Lowercase letters (a–f) above the bars represent the results of the multiple comparison analysis. Groups with different lowercase letters differ significantly (*p* < 0.05), whereas groups sharing at least one common lowercase letter are not significantly different according to the Kruskal–Wallis test followed by Bonferroni-adjusted post hoc comparisons.

Levels of TNF-α, IL-1β, and IL-6 in both the HIP and PFC were markedly higher in mice receiving CPX than in the control animals (*p* < 0.05). In contrast, the CPX + FLX group exhibited significantly lower levels of all three cytokines relative to the CPX group (*p* < 0.05) (Table 2, Figure 5).

In the HIP, CA treatment significantly reduced TNF-α, IL-1β, and IL-6 levels compared with the CPX group (*p* < 0.05). However, when compared with the CPX + FLX group, cytokine levels were comparable only in the CPX + CA100 group (*p* > 0.05). At the other doses, TNF-α and IL-6 levels remained significantly higher than those of the CPX + FLX group (*p* < 0.05), whereas IL-1β levels were similar across all CA-treated groups (*p* > 0.05).

In the PFC, CA administration significantly decreased TNF-α and IL-6 levels compared with the CPX group (*p* < 0.05). IL-1β levels were comparable to CPX in the CPX + CA50 group (*p* > 0.05) but were significantly reduced in the CPX + CA100 and CPX + CA200 groups (*p* < 0.05). Relative to the positive control group, TNF-α and IL-6 levels generally remained higher in the CA-treated groups (*p* < 0.05). Among these, the CPX + CA100 group showed cytokine levels most comparable to those of the CPX + FLX group. For IL-1β, the CPX + CA100 group was comparable to the positive control (*p* > 0.05), whereas the CPX + CA50 and CPX + CA200 groups exhibited significantly higher levels (*p* < 0.05).

### 3.3. Neurotransmitter Levels

**Figure 6:**
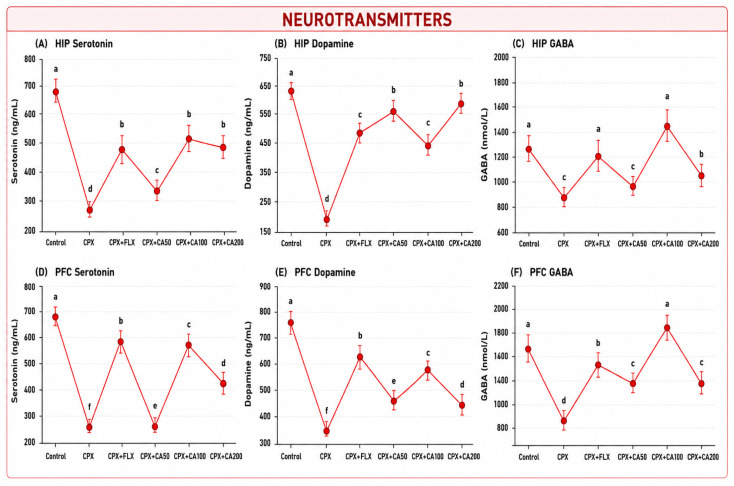
Effects of cinnamic acid (CA) on neurotransmitter levels in the hippocampus (HIP) and prefrontal cortex (PFC) of mice exposed to ciprofloxacin (CPX). (**A**) HIP serotonin, (**B**) HIP dopamine, (**C**) HIP GABA, (**D**) PFC serotonin, (**E**) PFC dopamine, and (**F**) PFC GABA levels. Data are presented as mean ± SD (*n* = 10). Lowercase letters (a–f) above the bars represent the results of the multiple comparison analysis. Groups with different lowercase letters differ significantly (*p* < 0.05), whereas groups sharing at least one common lowercase letter are not significantly different according to the Kruskal–Wallis test followed by Bonferroni-adjusted post hoc comparisons.

Neurotransmitter levels in the HIP and PFC revealed that CPX treatment significantly decreased serotonin, dopamine, and γ-aminobutyric acid (GABA) levels compared with the control group (*p* < 0.05). In contrast, the CPX + FLX group showed significantly higher levels of all three neurotransmitters relative to the CPX group in both regions (*p* < 0.05) (Table 3, Figure 6).

In the HIP, the CPX + CA100 group exhibited significant increases in serotonin, dopamine, and GABA levels compared with the CPX group (*p* < 0.05), reaching levels comparable to those of the CPX + FLX group (*p* > 0.05). In the CPX + CA50 group, serotonin levels remained significantly lower than those of the CA100, CA200, and positive control groups (*p* < 0.05). Dopamine levels in this group were comparable to those of the CA200 group (*p* > 0.05) but significantly higher than those of the CPX and CPX + FLX groups (*p* < 0.05).

In the CPX + CA200 group, serotonin levels were similar to those observed in the CA100 and CPX + FLX groups (*p* > 0.05) and significantly higher than those of the CPX group (*p* < 0.05). Dopamine levels were comparable to the CA50 group but remained significantly higher than those of the CPX and CPX + FLX groups (*p* < 0.05).

In the PFC, the CPX + CA50 group exhibited serotonin, dopamine, and GABA levels that were significantly lower than those of the positive control group but significantly higher than those of the CPX group (*p* < 0.05). The CPX + CA100 group showed significantly higher neurotransmitter levels than both the CPX and CA50 groups (*p* < 0.05), although values remained lower than those of the positive control group (*p* < 0.05). Similarly, the CPX + CA200 group demonstrated neurotransmitter levels significantly higher than those of the CPX and CA50 groups but lower than those of the CPX + FLX group (*p* < 0.05).

## 4. Discussion

In the present study, we comprehensively evaluated the effects of cinnamic acid (CA) on behavioral performance, oxidative stress status, pro-inflammatory cytokine responses, and neurotransmitter profiles in the hippocampus and prefrontal cortex in a ciprofloxacin (CPX)-induced experimental model of depression. Our findings demonstrated that CPX administration elicited depression-like behaviors, characterized by reduced social interaction and increased immobility in the forced swim and tail suspension tests. These behavioral alterations were accompanied by enhanced lipid peroxidation, suppression of antioxidant defense mechanisms, and marked elevations in TNF-α, IL-1β, and IL-6 levels in both brain regions. Furthermore, significant reductions in serotonergic, dopaminergic, and GABAergic neurotransmission were observed.

In contrast, CA administration effectively reversed these pathological alterations, with the 100 mg/kg dose producing the most pronounced therapeutic effects. Notably, treatment with CA at 100 mg/kg produced marked improvements in behavioral outcomes and exhibited a therapeutic profile largely comparable to that of the positive control group (CPX + FLX) with respect to oxidative stress markers, pro-inflammatory cytokine levels, and neurotransmitter balance. These findings suggest that activation of the neuroinflammatory axis plays a pivotal role in the depression-like phenotype induced by CPX. Indeed, the ability of CA at 100 mg/kg to suppress hippocampal TNF-α, IL-1β, and IL-6 levels to values comparable to those achieved with fluoxetine, together with concurrent restoration of serotonin, dopamine, and GABA levels and significant improvements in behavioral parameters—including social interaction, behavioral despair, and anhedonia—highlights a strong association between anti-inflammatory effects and behavioral recovery. Collectively, these observations indicate that the antidepressant-like effects of CA may not be solely attributable to its antioxidant capacity but may also involve multifaceted and region-specific mechanisms that modulate the interplay between inflammation and neurotransmitter systems. Previous experimental studies have demonstrated that various natural bioactive compounds can attenuate pro-inflammatory cytokine responses and reduce tissue damage in experimental disease models [30]. These findings further support the therapeutic potential of naturally derived compounds in conditions characterized by inflammation and oxidative stress.

Furthermore, CPX administration consistently induced a depression-like phenotype characterized by reduced social interaction, increased immobility in the forced swim and tail suspension tests, and decreased sucrose preference. These behavioral alterations are indicative of anhedonia, social withdrawal, and behavioral despair, thereby supporting the validity of the CPX-induced experimental depression model.

The FST is a well-established and widely used experimental paradigm for assessing depression-like behaviors and is considered to reflect behavioral despair. Increased immobility time in this test is generally interpreted as an indicator of passive coping strategies in response to stress [1,31]. Similarly, the TST is recognized as a reliable behavioral model for evaluating passive stress-coping behavior and depressive-like phenotypes in rodents [32].

The SPT is one of the principal behavioral paradigms used to assess anhedonia and reflects dysfunction of the brain reward system. Reduced sucrose consumption is widely accepted as an indicator of impaired hedonic capacity [33].

In addition, the DSE test represents an important behavioral approach for evaluating social motivation, social withdrawal, and mood-related alterations. Decreased social interaction observed in this test is considered to reflect social withdrawal behaviors commonly associated with depressive states [24,34].

Consistent with these established paradigms, CPX administration in the present study produced a depression-like phenotype characterized by impaired social interaction, the development of anhedonia, passive stress-coping behaviors, and increased behavioral despair. These findings are in agreement with previous reports demonstrating that CPX exposure induces experimental depression-like states in rodents [1,11,12,13].

The reduction in swimming activity observed following CPX administration, together with the increased immobility time in the tail suspension test, appears to be closely associated with decreased serotonin and dopamine levels in the central nervous system [1,35]. Serotonin is one of the principal neurotransmitters involved in mood regulation and plays critical roles in synaptic plasticity, stress responsiveness, and emotional behavior [36]. Alterations in serotonergic and dopaminergic signaling, particularly within brain regions that govern affective processing such as the hippocampus and prefrontal cortex, have been strongly implicated in the pathophysiology of depression through disruption of synaptic plasticity [37,38].

Consistent with this view, suppression of serotonergic activity in experimental depression models has been closely linked to increased behavioral despair and reduced motivational responses [1,11,13]. In the present study, CPX treatment significantly decreased serotonin and dopamine levels in both the HIP and PFC, which coincided with shortened swimming time, prolonged immobility, and an overall passive behavioral profile. Moreover, reduced dopamine levels were accompanied by pronounced anhedonic behavior, as demonstrated by decreased sucrose preference.

Anhedonia represents a key behavioral feature of depression and is commonly linked to impairments in reward processing and motivational drive. Reduced sucrose consumption in the SPT is therefore considered to reflect insufficient dopamine signaling in reward-related brain regions, including the PFC [39,40,41].

In addition to monoaminergic systems, the GABAergic network plays a critical role in emotional regulation and cortical excitability. GABAergic interneurons in the hippocampus and prefrontal cortex maintain neural stability by limiting excessive neuronal activity [42]. Reduced GABAergic tone has been reported to weaken inhibitory control over serotonergic and dopaminergic circuits, thereby contributing to impairments in reward processing, motivation, and social behavior.

Taken together, the decreases in serotonin, dopamine, and GABA detected in both brain regions appear to be associated with the social impairment, behavioral despair, and anhedonia observed following CPX exposure.

Several theoretical frameworks have been proposed to explain the pathophysiological mechanisms underlying depression, among which the monoaminergic neurotransmitter hypothesis, hypothalamic–pituitary–adrenal (HPA) axis dysregulation, neuroinflammation, and oxidative stress are widely recognized as key contributors to both the development and persistence of the disorder [43].

In this context, the present study comprehensively evaluated not only neurotransmitter systems (serotonin, dopamine, and GABA) but also cytokines (TNF-α, IL-1β, and IL-6) and oxidative stress markers (MDA, GSH, and CAT), all of which are closely implicated in the neurobiological basis of depression.

Recent evidence has increasingly demonstrated that elevated pro-inflammatory cytokine levels are significantly associated with the severity of depressive symptoms [44,45]. In experimental depression models, increased TNF-α, IL-1β, and IL-6 levels in the PFC and HIP have been shown to enhance hypothalamic–pituitary–adrenal (HPA) axis activity, thereby contributing to cognitive impairment, mood disturbances, and depression-like behaviors [45]. Pro-inflammatory mediators such as TNF-α and IL-1β are known to trigger microglial activation, reduce neuroplasticity, and disrupt neuronal circuits that are critical for mood regulation [23]. Moreover, elevated IL-1β levels have been reported to induce astrocyte activation, which may subsequently alter neurotransmitter release and promote anxiety- and depression-like behaviors [46]. Experimental studies further indicate that IL-1β infusion activates hypothalamic astrocytes and elicits anxiety-like responses, whereas inhibition of astrocytic activity attenuates these behaviors, partly through modulation of astrocyte-derived GABA release [47].

Astrocytes are recognized as an important source of IL-6 production within the CNS. Although IL-6 may exert neuroprotective actions under physiological conditions, excessive production has been associated with neurodegenerative changes [44]. Studies employing chronic mild stress models have demonstrated that increased IL-6 levels trigger depression-like behaviors. Consistently, elevated IL-6 concentrations have been detected in both plasma and cerebrospinal fluid of patients diagnosed with major depressive disorder and in individuals with a history of suicide attempts [48]. Furthermore, higher plasma IL-6 levels have been observed in patients who exhibit resistance to treatment with SSRIs or serotonin–noradrenaline reuptake inhibitors (SNRIs). IL-6 concentrations were also shown to remain persistently elevated in patients with major depressive disorder who fail to respond to therapy. Collectively, these findings suggest that IL-6 may play a critical role in the pathophysiology of treatment-resistant depression [49]. On the other hand, increased pro-inflammatory cytokine activity promotes the diversion of tryptophan—the primary precursor of serotonin (5-HT)—toward the kynurenine pathway. This metabolic shift reduces synaptic 5-HT bioavailability, thereby impairing serotonergic neurotransmission [50].

Inflammatory cytokines can enhance NO signaling, leading to diminished BH_4_ availability. Since tyrosine hydroxylase, the key regulatory enzyme in dopamine production, requires BH_4_ for its activity, this mechanism may contribute to reduced dopamine synthesis. This reduction may consequently impair the biosynthesis of both dopamine and norepinephrine [47]. In addition, TNF-α has been reported to suppress serotonergic neurotransmission by enhancing serotonin reuptake. Moreover, TNF-α inhibits glutamine synthetase activity, disrupts GABAergic transmission, decreases glutamate reuptake, and adversely affects synaptic plasticity mechanisms, including long-term potentiation (LTP) [44]. CPX exposure has been shown to suppress mitochondrial DNA (mtDNA) replication, leading to reduced mtDNA content. This reduction has been proposed to compromise the functional integrity of the electron transport chain, ultimately resulting in mitochondrial dysfunction and excessive reactive oxygen species (ROS) production [51].

Indeed, CPX has been reported to increase the formation of ROS such as hydroxyl radicals (OH), superoxide anions (O_2_^−^), and hydrogen peroxide (H_2_O_2_) in brain tissue, which may exert detrimental effects on the central nervous system [1]. Due to its high oxygen consumption and lipid-rich composition, brain tissue is particularly susceptible to oxidative damage. In this context, MDA, the end product of lipid peroxidation, and CAT, a key antioxidant enzyme responsible for ROS detoxification, are widely used biochemical markers for assessing oxidative stress [43]. Several studies have reported that CPX administration increases MDA levels while decreasing GSH content and CAT activity in brain tissue [1,13,20]. The biochemical findings obtained in the current study revealed marked alterations in MDA, GSH, and CAT levels following CPX exposure, further supporting the involvement of oxidative stress-related mechanisms. The elevation in CAT activity observed in CPX-treated animals may represent an adaptive antioxidant response to increased oxidative challenge. The observed increase in CAT activity in the CPX group may reflect a compensatory or adaptive antioxidant response to elevated oxidative burden. However, the marked depletion of GSH suggests an overall disruption of redox homeostasis. In contrast, FLX and CA treatments partially restored redox balance by reducing MDA levels and increasing GSH concentrations. Elevated ROS production is also known to activate intracellular signaling cascades, particularly pro-inflammatory transcription factors such as NF-κB, thereby promoting the synthesis and release of pro-inflammatory cytokines including TNF-α, IL-1β, and IL-6 [3].

In the present study, CPX administration is presumed to have increased oxidative stress levels in brain tissue by enhancing the generation of ROS, including H_2_O_2_, OH, and O_2_^−^. This excessive ROS production may have consequently triggered neuroinflammatory processes. ROS accumulation associated with CPX exposure may activate NF-κB signaling pathways, thereby upregulating the expression of pro-inflammatory cytokines such as TNF-α, IL-1β, and IL-6. By amplifying microglial activation, increased cytokine production may support the propagation and persistence of neuroinflammation in the CNS.

Elevated oxidative stress is thought to suppress serotonergic neurotransmission by redirecting tryptophan metabolism toward the kynurenine pathway, thereby reducing serotonin availability. Concurrently, oxidative stress may impair dopaminergic neurotransmission through the depletion of tetrahydrobiopterin (BH_4_), an essential cofactor required for catecholamine synthesis. In addition, the inhibition of glutamate reuptake and the attenuation of GABAergic inhibitory tone mediated by TNF-α and IL-1β disrupt the excitatory–inhibitory balance within neural circuits. The combined effects of these molecular disturbances may impair synaptic adaptability within neural circuits responsible for emotional regulation, particularly those involving the HIP and PFC, thereby facilitating depression-like behaviors [43].

Taken together, the present findings indicate that CPX adversely affects central nervous system function through an integrated oxidative stress–neuroinflammation–neurotransmitter dysfunction axis, which may underlie the development of depression-like phenotypes. These results not only contribute to a better understanding of the mechanisms underlying CPX-associated neuropsychiatric effects but also suggest that targeting inflammatory pathways and oxidative stress may represent a promising therapeutic strategy.

FLX primarily exerts its therapeutic effects by enhancing serotonergic neurotransmission. Beyond its classical monoaminergic mechanism of action, accumulating evidence indicates that FLX also possesses significant antioxidant properties, which are associated with reduced lipid peroxidation and the reinforcement of endogenous antioxidant defense systems [52]. Several experimental studies have demonstrated that fluoxetine decreases oxidative stress markers while concomitantly increasing the activity of key antioxidant enzymes [53,54]. In addition, FLX may modulate neuroinflammatory processes by interfering with NF-κB-dependent signaling, leading to a decline in TNF-α, IL-1β, and IL-6 production [55]. Importantly, these findings suggest that FLX not only modulates monoaminergic transmission but also exerts pronounced anti-inflammatory effects through the inhibition of pro-inflammatory cytokine production, highlighting its broader neuroprotective and immunomodulatory potential [55].

The findings of this study indicate that CPX exposure was associated with depression-like behavioral alterations accompanied by increased oxidative stress and neuroinflammatory activity. In contrast, FLX treatment significantly reversed these detrimental effects. The CPX-induced elevations in TNF-α, IL-1β, and IL-6 levels were markedly attenuated following FLX administration, supporting the anti-inflammatory properties of the drug. Furthermore, FLX reduced oxidative stress markers, enhanced antioxidant defense capacity, and restored the balance of serotonergic, dopaminergic, and GABAergic neurotransmission.

CA and its derivatives have emerged as important targets of pharmacological research due to their widespread occurrence in natural sources, low-toxicity profiles, and structural diversity. A variety of pharmacological effects have been attributed to these compounds, including anti-inflammatory [56], antioxidant [57], antitumor [58], hypoglycemic [59], and antidepressant properties [60]. Notably, accumulating evidence indicates that CA derivatives may confer neuroprotective benefits by mitigating neuroinflammatory processes, particularly in the context of neurodegenerative disorders [22]. CA administration has been reported to increase serotonin and GABA levels in the hypothalamus of rats subjected to an experimental insomnia model [61]. Moreover, in a rat model of Parkinson’s disease, CA administered at a dose of 100 mg/kg exhibited neuroprotective properties within the nigrostriatal dopaminergic system, attenuating dopaminergic neuronal loss and preserving striatal dopamine levels. In addition to maintaining dopaminergic tone, CA was shown to suppress microglial activation, reduce the expression of pro-inflammatory cytokines such as TNF-α and IL-1β, and decrease oxidative stress markers [62].

Interestingly, CAT activity was significantly elevated in the CPX-treated group despite the presence of marked oxidative stress. This increase may represent a compensatory adaptive response aimed at counteracting excessive reactive oxygen species production. Although CA treatment reduced lipid peroxidation, particularly at the 100 mg/kg dose, CAT activity remained partially elevated in the CA200 group. This finding suggests that antioxidant enzyme responses may not necessarily normalize in parallel with oxidative damage markers and could reflect the continued adaptive regulation of endogenous antioxidant defense mechanisms.

Similarly, CA at doses of 100 and 200 mg/kg has been shown to significantly ameliorate depression-related behavioral impairments in an experimental depression model. These beneficial effects were attributed to the suppression of pro-inflammatory cytokines and the restoration of redox homeostasis [22]. Furthermore, D-amino acid-containing CA derivatives were reported to exert pronounced anti-neuroinflammatory effects in a lipopolysaccharide (LPS)-induced neuroinflammation model. These derivatives suppressed nitric oxide (NO) and IL-1β production, reversed pathological alterations in the hippocampus, and consequently improved working memory and spatial cognitive performance [63]. Consistent with these findings, CA treatment in diabetic rats reduced elevated MDA levels while increasing depleted GSH and CAT levels in brain tissue, thereby strengthening antioxidant defense systems and improving memory function [19]. Belonging to the phenolic acid group, CA exhibits antioxidant activity through its ability to interact with reactive oxygen species and other oxidizing molecules [60]. This property may contribute to the preservation of cellular homeostasis under conditions of oxidative challenge. In addition, CA has been reported to regulate inflammatory signaling cascades, including NF-κB-associated pathways, thereby limiting neuroinflammatory processes [64].

In the present study, CA treatment attenuated the depression-like phenotype induced by CPX, with the 100 mg/kg dose showing the most consistent behavioral and biochemical benefits. Behavioral assessments demonstrated that CA, particularly at the 100 mg/kg dose, exerted marked efficacy by improving social interaction parameters. Furthermore, CA reduced immobility time in the FST, reflecting an antidepressant-like profile, and significantly ameliorated the reduction in sucrose preference, a well-established indicator of anhedonia.

At the biochemical level, CA administration at doses of 100 and 200 mg/kg significantly suppressed CPX-induced lipid peroxidation in both the hippocampus and prefrontal cortex and partially restored antioxidant defense components. Nevertheless, the present findings suggest that the elevation of pro-inflammatory cytokines plays a predominant role in the development of the depression-like phenotype, and that modulation of this inflammatory axis represents a key determinant of behavioral recovery. Notably, the 100 mg/kg CA dose exhibited an effect profile most comparable to that of the positive control, FLX, effectively reducing CPX-induced increases in TNF-α, IL-1β, and IL-6 levels, thereby indicating robust suppression of CPX-triggered neuroinflammatory responses.

Importantly, this cytokine attenuation appears to have direct neurochemical and behavioral correlates. Compared with other CA doses, 100 mg/kg more effectively reversed CPX-induced reductions in serotonin, dopamine, and GABA levels, particularly within the hippocampus. This neurochemical normalization is likely to support monoaminergic and GABAergic neurotransmission along the hippocampal–prefrontal cortex (HIP–PFC) circuitry, which is critically involved in mood regulation, thereby contributing to more pronounced improvements in behavioral parameters, including social interaction, behavioral despair, and anhedonia. The more pronounced efficacy observed at the 100 mg/kg dose suggests that this dose may provide the optimal therapeutic benefit of cinnamic acid under the present experimental conditions.

## 5. Limitations and Future Directions

While behavioral and biochemical parameters were comprehensively assessed, the mechanistic basis of cinnamic acid activity was not fully elucidated. In particular, molecular signaling pathways associated with neuroinflammation and oxidative stress, such as NF-κB and Nrf2, were not directly analyzed. In addition, the absence of a cinnamic acid-only control group precluded the evaluation of the intrinsic effects of cinnamic acid under normal physiological conditions. Furthermore, the present study focused on a single experimental model of ciprofloxacin-induced depression; therefore, the generalizability of these findings to other models of depression may be limited. Future studies integrating molecular pathway analyses, gene expression profiling, cinnamic acid-only treatment groups, and translational experimental models are warranted to further elucidate the therapeutic potential of cinnamic acid and its possible clinical relevance in the management of depression.

## 6. Conclusions

Administration of CA reduced the behavioral and neurochemical alterations associated with CPX exposure in mice. Beneficial effects were observed across all tested doses, and the most pronounced overall improvement was observed with the 100 mg/kg dose. These changes were accompanied by improvements in oxidative stress markers, inflammatory mediators, and neurotransmitter profiles in the hippocampus and prefrontal cortex. Taken together, the findings indicate that CA may help counteract CPX-related neuropsychiatric disturbances through the modulation of redox balance, inflammatory activity, and neurotransmitter function. The cellular and molecular basis of these findings warrants further investigation.

## Figures and Tables

**Table 1:** Levels of MDA, GSH, and CAT in PFC and HIP tissues following CPX administration.

	MDA (nmol/mL)	GSH (mg/L)	CAT (ng/mL)
HIP			
Control	7.34 ± 0.53 ^d^	32.67 ± 1.53 ^a^	40.40 ± 0.35 ^f^
CPX	12.41 ± 0.00 ^a^	14.11 ± 1.35 ^e^	94.13 ± 0.61 ^a^
CPX + FLX	9.84 ± 0.24 ^c^	30.33 ± 0.67 ^b^	43.87 ± 0.83 ^e^
CPX + CA50	10.98 ± 0.49 ^b^	16.11 ± 1.17 ^d^	85.47 ± 1.01 ^b^
CPX + CA100	9.21 ± 1.00 ^c^	25.67 ± 0.58 ^c^	45.67 ± 0.83 ^d^
CPX + CA200	9.38 ± 0.27 ^c^	27.44 ± 0.51 ^c^	73.40 ± 0.72 ^c^
	*p* < 0.001 H = 30.8	*p* < 0.001 H = 32.5	*p* < 0.05 H = 17.3
PFC			
Control	7.41 ± 0.76 ^d^	48.44 ± 1.02 ^a^	92.80 ± 2.80 ^d^
CPX	13.30 ± 0.55 ^a^	26.11 ± 0.51 ^e^	136.53 ± 2.20 ^a^
CPX + FLX	8.30 ± 1.00 ^d^	36.44 ± 0.77 ^b^	95.47 ± 2.60 ^d^
CPX + CA50	11.23 ± 0.20 ^b^	30.22 ± 1.02 ^d^	115.73 ± 3.72 ^b^
CPX + CA100	7.72 ± 0.35 ^d^	33.00 ± 0.33 ^c^	101.20 ± 4.18 ^c^
CPX + CA200	9.93 ± 0.75 ^c^	36.44 ± 1.02 ^b^	95.13 ± 1.81 ^d^
	*p* < 0.001 H = 33.8	*p* < 0.001 H = 34.1	*p* < 0.001 H = 27.5

Values marked with distinct superscript letters in a given column represent statistically different groups (*p* < 0.05). Data are shown as mean ± standard deviation for each group (*n* = 10). CPX, ciprofloxacin; CA, cinnamic acid; FLX, fluoxetine; MDA, malondialdehyde; GSH, glutathione; CAT, catalase; HIP, hippocampus; PFC, prefrontal cortex.

**Table 2:** TNF-α, IL-1β, and IL-6 concentrations in HIP and PFC tissues following CPX administration.

	TNF-α (ng/L)	IL-1β (pg/L)	IL-6 (ng/L)
HIP			
Control	65.55 ± 1.02 ^e^	875.13 ± 3.96 ^b^	48.67 ± 1.53 ^e^
CPX	191.85 ± 3.23 ^a^	1508.51 ± 52.04 ^a^	147.33 ± 3.06 ^a^
CPX + FLX	103.70 ± 4.30 ^d^	920.12 ± 72.98 ^b^	75.33 ± 1.53 ^d^
CPX + CA50	165.55 ± 1.88 ^b^	929.46 ± 17.97 ^b^	127.33 ± 7.02 ^b^
CPX + CA100	106.27 ± 5.45 ^d^	872.80 ± 25.34 ^b^	78.33 ± 2.08 ^d^
CPX + CA200	123.70 ± 3.03 ^c^	1072.80 ± 407.12 ^b^	85.33 ± 1.53 ^c^
	*p* < 0.001 H = 33.3	*p* < 0.005 H = 21.4	*p* < 0.001 H = 33.6
PFC			
Control	179.97 ± 3.18 ^f^	821.61 ± 32.73 ^c^	55.33 ± 1.53 ^f^
CPX	354.76 ± 3.97 ^a^	1444.23 ± 114.75 ^a^	159.67 ± 2.52 ^a^
CPX + FLX	205.21 ± 2.58 ^e^	886.13 ± 25.16 ^c^	83.67 ± 1.53 ^e^
CPX + CA50	323.85 ± 3.92 ^b^	1370.42 ± 120.39 ^a^	141.67 ± 3.79 ^b^
CPX + CA100	216.88 ± 3.87 ^d^	848.32 ± 46.82 ^c^	90.67 ± 1.53 ^d^
CPX + CA200	236.12 ± 1.84 ^c^	1127.12 ± 11.23 ^b^	121.00 ± 1.00 ^c^
	*p* < 0.001 H = 34.0	*p* < 0.001 H = 31.3	*p* < 0.001 H = 34.1

Values marked with distinct superscript letters in a given column represent statistically different groups (*p* < 0.05). Data are shown as mean ± standard deviation for each group (*n* = 10). CPX, ciprofloxacin; CA, cinnamic acid; FLX, fluoxetine; TNF-α, tumor necrosis factor-alpha; IL-1β, interleukin-1 beta; IL-6, interleukin-6; HIP, hippocampus; PFC, prefrontal cortex.

**Table 3:** Neurotransmitter levels (serotonin, dopamine, and GABA) in the HIP and PFC in the CPX-induced depression model.

	Serotonin (ng/mL)	Dopamine (ng/mL)	GABA (nmol/L)
HIP			
Control	645.36 ± 3.88 ^a^	651.43 ± 4.99 ^a^	1210.45 ± 202.73 ^a^
CPX	280.06 ± 9.43 ^d^	202.15 ± 3.52 ^d^	875.48 ± 160.59 ^c^
CPX + FLX	433.69 ± 11.69 ^b^	465.81 ± 30.41 ^c^	1114.25 ± 54.23 ^a^
CPX + CA50	305.00 ± 0.88 ^c^	552.46 ± 7.65 ^b^	967.61 ± 51.83 ^c^
CPX + CA100	446.27 ± 13.58 ^b^	463.19 ± 6.82 ^c^	1312.69 ± 246.47 ^a^
CPX + CA200	437.03 ± 11.28 ^b^	567.46 ± 15.32 ^b^	1023.29 ± 131.43 ^b^
	*p* < 0.001 H = 30.8	*p* < 0.001 H = 32.5	*p* < 0.05 H = 17.3
PFC			
Control	704.30 ± 5.42 ^a^	793.83 ± 11.33 ^a^	1650.51 ± 140.37 ^a^
CPX	219.75 ± 5.42 ^f^	263.46 ± 6.43 ^f^	1014.94 ± 38.22 ^d^
CPX + FLX	651.87 ± 30.86 ^b^	586.48 ± 14.39 ^b^	1419.82 ± 194.55 ^b^
CPX + CA50	263.84 ± 14.52 ^e^	382.48 ± 12.61 ^e^	1269.35 ± 41.70 ^c^
CPX + CA100	590.21 ± 3.22 ^c^	503.10 ± 7.52 ^c^	1864.76 ± 135.70 ^a^
CPX + CA200	426.12 ± 8.98 ^d^	452.53 ± 7.63 ^d^	1313.80 ± 201.48 ^c^
	*p* < 0.001 H = 33.8	*p* < 0.001 H = 34.1	*p* < 0.001 H = 27.5

Values marked with distinct superscript letters in a given column represent statistically different groups (*p* < 0.05). Data are shown as mean ± standard deviation for each group (*n* = 10). CPX, ciprofloxacin; CA, cinnamic acid; FLX, fluoxetine; GABA, gamma-aminobutyric acid; HIP, hippocampus; PFC, prefrontal cortex.

## Data Availability

The data that support the findings of this study are available from the corresponding author upon reasonable request.

## References

[1] Salama A., Mahmoud H.A.A., Kandeil M.A., Khalaf M.M. (2021). Neuroprotective role of camphor against ciprofloxacin induced depression in rats: Modulation of Nrf-2 and TLR4. Immunopharmacol. Immunotoxicol..

[2] Abdel-Rasoul A.A., Saleh N.A., Hosny E.N., El-Gizawy M.M., Ibrahim E.A. (2023). Cardamom oil ameliorates behavioral and neuropathological disorders in a rat model of depression induced by reserpine. J. Ethnopharmacol..

[3] Correia A.S., Cardoso A., Vale N. (2023). Oxidative stress in depression: The link with the stress response, neuroinflammation, serotonin, neurogenesis and synaptic plasticity. Antioxidants.

[4] Hassamal S. (2023). Chronic stress, neuroinflammation, and depression: An overview of pathophysiological mechanisms and emerging anti-inflammatories. Front. Psychiatry.

[5] Sălcudean A. (2025). Neuroinflammation—A crucial factor in depression. Biomolecules.

[6] Ren S., Guo Z., Zhang J., He Y., Sun Z., Yang J. (2025). Transcriptomic alterations in the hippocampus and prefrontal cortex of rats with chronic unpredictable stress induced by low-intensity pulsed ultrasound. Mol. Neurobiol..

[7] Tang Q., Li B., Sun W., Jiang S., Liu Y., Xu X., Sun D., Qu M. (2025). Effects of Shenqi Jieyu formula on perfusion and brain structure in the hippocampus and prefrontal cortex of postpartum depressed rats: A functional magnetic resonance imaging study. Brain Behav. Immun. Integr..

[8] Ruggiero R.N., Rossignoli M.T., Marques D.B., de Sousa B.M., Romcy-Pereira R.N., Lopes-Aguiar C., Leite J.P. (2021). Neuromodulation of hippocampal-prefrontal cortical synaptic plasticity and functional connectivity: Implications for neuropsychiatric disorders. Front. Cell Neurosci..

[9] Maria Michel T., Pulschen D., Thome J. (2012). The role of oxidative stress in depressive disorders. Curr. Pharm. Des..

[10] Wan Z., Cai S., Mo H. (2026). Mediation roles of oxidative stress, inflammation, and insulin resistance biomarkers in the sitting time-depression association among US adults. J. Affect. Disord..

[11] Rahman H., Anggadiredja K., Sasongko L. (2025). Mechanisms of oral ciprofloxacin-induced depressive-like behavior and the potential benefit of lactulose: A correlation analysis. Toxicol. Rep..

[12] Khalaf M.M., Mahmoud H.M., Kandeil M.A., Mahmoud H.A., Salama A.A. (2024). Fumaric acid protects rats from ciprofloxacin-provoked depression through modulating TLR4, Nrf-2, and p190-rho GTP. Drug Chem. Toxicol..

[13] Ilgin S., Can O.D., Atli O., Ucel U.I., Sener E., Guven I. (2015). Ciprofloxacin-induced neurotoxicity: Evaluation of possible underlying mechanisms. Toxicol. Mech. Methods.

[14] Filipović D., Turck C.W. (2025). Prefrontal cortex molecular signatures of chronically socially isolated rats and their response to fluoxetine treatment. Mol. Neurobiol..

[15] Pérez M.G.S., Jiménez Y., Bagán L., Bagán J.V. (2025). Oral side effects of fluoxetine in patients with depressive disorder: A systematic review. Med. Oral Patol. Oral Cir. Bucal.

[16] Chandra S., Roy A., Jana M., Pahan K. (2019). Cinnamic acid activates PPARα to stimulate lysosomal biogenesis and lower amyloid plaque pathology in an Alzheimer’s disease mouse model. Neurobiol. Dis..

[17] Sova M. (2012). Antioxidant and antimicrobial activities of cinnamic acid derivatives. Mini Rev. Med. Chem..

[18] Kumar A., Khan F., Saikia D. (2022). Phenolic Compounds and Their Biological and Pharmaceutical Activities. The Chemistry Inside Spices & Herbs: Research and Development.

[19] Hemmati A.A., Alboghobeish S., Ahangarpour A. (2018). Effects of cinnamic acid on memory deficits and brain oxidative stress in streptozotocin-induced diabetic mice. Korean J. Physiol. Pharmacol..

[20] Rawi S.M., Mourad I.M., Arafa N.M., Alazabi N.I. (2011). Effect of ciprofloxacin and levofloxacin on some oxidative stress parameters in brain regions of male albino rats. Afr. J. Pharm. Pharmacol..

[21] Atallah A.H., Masaoodi N.N.A., Obaid K.H. (2022). Evaluation of the effect of ciprofloxacin on some cytokines in mice. J. Pharm. Negat. Results.

[22] Zhuo R., Cheng X., Luo L., Yang L., Zhao Y., Zhou Y., Peng L., Jin X., Cui L., Liu F. (2022). Cinnamic acid improved lipopolysaccharide-induced depressive-like behaviors by inhibiting neuroinflammation and oxidative stress in mice. Pharmacology.

[23] Alacabey N.A., Coşkun D., Çeribaşi S., Ateşşahin A. (2025). Effects of boron on learning and behavioral disorders in rat autism model induced by intracerebroventricular propionic acid. Biol. Trace Elem. Res..

[24] Detke M.J., Lucki I. (1995). Detection of serotonergic and noradrenergic antidepressants in the rat forced swimming test. Psychopharmacology.

[25] Rickels M.J., Lucki M. (1997). Active behaviors in the rat forced swimming test differentially produced by serotonergic and noradrenergic antidepressants. Psychopharmacology.

[26] Steru L., Chermat R., Thierry B., Simon P. (1985). The tail suspension test: A new method for screening antidepressants in mice. Psychopharmacology.

[27] Tiwari V., Kuhad A., Chopra K. (2011). Suppression of neuro-inflammatory signaling pathway by curcumin attenuates chronic stress-induced depressive-like behavior in rats. Behav. Brain Res..

[28] Mao Q.Q., Ip S.P., Ko K.M., Tsai S.H., Che C.T. (2012). Peony glycosides protect against corticosterone-induced depression-like behaviors in mice through neuroprotective mechanisms. J. Ethnopharmacol..

[29] Baghaei Naeini F., Hassanpour S., Asghari A. (2023). Resveratrol exerts anxiolytic-like effects through anti-inflammatory and antioxidant activities in rats exposed to chronic social isolation. Behav. Brain Res..

[30] Akman N., Yaman T., Kömüroğlu A.U., Çalışır M. (2026). Effects of bee bread (Perga) on pro-inflammatory cytokine levels and histopathological alterations in the liver and kidneys of streptozotocin-induced diabetic rats. Biology.

[31] Della Valle A., De Carlo S., Sonsini G., Pilati S., Perali A., Ubaldi M., Ciccocioppo R. (2025). Machine learning-based model for behavioural analysis in rodents applied to the forced swim test. Sci. Rep..

[32] Trunnell E.R., Baines J., Farghali S., Jackson T., Jayne K., Smith R., Stibbe T. (2024). The need for guidance in antidepressant drug development: Revisiting the role of the forced swim test and tail suspension test. Regul. Toxicol. Pharmacol..

[33] Markov D.D. (2022). Sucrose preference test as a measure of anhedonic behavior in a chronic unpredictable mild stress model of depression: Outstanding issues. Brain Sci..

[34] Mohseni F., Rafaiee R. (2025). Opinion: Ethical challenges in depression research: The tail suspension test, the forced swim test, and alternative behavioral models. J. Am. Assoc. Lab. Anim. Sci..

[35] Atiyah I.A., Niyomdecha S., Cheaha D. (2025). Neural oscillations in the nucleus accumbens–dorsal hippocampal circuits and behavioral effects of acute fluoxetine administration during the tail suspension test in mice. Exp. Brain Res..

[36] Cui L., Li S., Wang S., Wu X., Liu Y., Yu W., Wang Y., Tang Y., Xia M., Li B. (2024). Major depressive disorder: Hypothesis, mechanism, prevention and treatment. Signal Transduct. Target. Ther..

[37] Xu X., Zheng C., An L., Wang R., Zhang T. (2016). Effects of dopamine and serotonin systems on modulating neural oscillations in hippocampus-prefrontal cortex pathway in rats. Brain Topogr..

[38] Çalışkan H., Cihan K.H., Koçak S., Karabulut G., Nalçacı E. (2025). Hypoxia disrupted serotonin levels in the prefrontal cortex and striatum, leading to depression-like behavior. Biology.

[39] Primo M.J., Fonseca-Rodrigues D., Almeida A., Teixeira P.M., Pinto-Ribeiro F. (2023). Sucrose preference test: A systematic review of protocols for the assessment of anhedonia in rodents. Eur. Neuropsychopharmacol..

[40] Berrio J.P., Hestehave S., Kalliokoski O. (2024). The sucrose preference threshold for anhedonia in stress models. Transl. Psychiatry.

[41] Scheggi S. (2024). Still controversial issues on assessing anhedonia in experimental modeling of depression. Transl. Psychiatry.

[42] Lüscher B., Möhler H. (2019). Brexanolone, a neurosteroid antidepressant, vindicates the GABAergic deficit hypothesis of depression and may foster resilience. F1000Research.

[43] Malhi G.S., Mann J.J. (2018). Depression. Lancet.

[44] Akman N., Kömüroğlu A. (2026). Effect of ciprofloxacin on some oxidative stress parameters and TNF-α, IL-6 and BDNF in diabetic rats with urinary tract infection induced by *Escherichia coli*. Indian J. Pharm. Educ. Res..

[45] Wang W., Yang J., Xu J., Yu H., Liu Y., Wang R., Ho R.C., Ho C.S., Pan F. (2022). Effects of high-fat diet and chronic mild stress on depression-like behaviors and levels of inflammatory cytokines in the hippocampus and prefrontal cortex of rats. Neuroscience.

[46] Czéh B., Welt T., Fischer A.K., Erhardt A., Schmitt W., Müller M.B., Toschi N., Fuchs E., Keck M.E. (2002). Chronic psychosocial stress and concomitant repetitive transcranial magnetic stimulation: Effects on stress hormone levels and adult hippocampal neurogenesis. Biol. Psychiatry.

[47] Rhie S.J., Jung E.Y., Shim I. (2020). The role of neuroinflammation on pathogenesis of affective disorders. J. Exerc. Rehabil..

[48] Shim H.S., Park H.J., Woo J., Lee C.J., Shim I. (2019). Role of astrocytic GABAergic system on inflammatory cytokine-induced anxiety-like behavior. Neuropharmacology.

[49] Akman N., Kömüroğlu A.U., Çibuk S., Altındağ F., Yılmaz O., Ateşşahin A. (2026). Effects of infliximab in a propionic acid-induced experimental autism rat model. Biomedicines.

[50] Dellink A., Vanderhaegen G., Coppens V., Ryan K.M., McLoughlin D.M., Kruse J., van Exel E., van Diermen L., Belge J.-B., Aarsland T.I.M. (2025). Inflammatory markers associated with electroconvulsive therapy response in patients with depression: A meta-analysis. Neurosci. Biobehav. Rev..

[51] Hangas A., Aasumets K., Kekäläinen N.J., Paloheinä M., Pohjoismäki J.L., Gerhold J.M., Goffart S. (2018). Ciprofloxacin impairs mitochondrial DNA replication initiation through inhibition of topoisomerase 2. Nucleic Acids Res..

[52] Yan L., Xu X., He Z., Wang S., Zhao L., Qiu J., Wang D., Gong Z., Qiu X., Huang H. (2020). Antidepressant-Like Effects and Cognitive Enhancement of Coadministration of Chaihu Shugan San and Fluoxetine: Dependent on the BDNF-ERK-CREB Signaling Pathway in the Hippocampus and Frontal Cortex. BioMed Res. Int..

[53] Khanzode S.D., Dakhale G.N., Khanzode S.S., Saoji A., Palasodkar R. (2003). Oxidative damage and major depression: The potential antioxidant action of selective serotonin reuptake inhibitors. Redox Rep..

[54] Alboni S., Benatti C., Montanari C., Tascedda F., Brunello N. (2013). Chronic antidepressant treatments resulted in altered expression of genes involved in inflammatory responses in the rat hippocampus. Eur. Neuropsychopharmacol..

[55] Hosseinirezaabad S., Motaghi S., Dogani M., Teimouri M. (2025). Antidepressant effects of fluoxetine: Upregulation of connexin 36 and 43 in the hippocampus, prefrontal cortex, and amygdala. Mol. Biol. Rep..

[56] Theodoridis K., Charissopoulos E., Tsioumela D., Pontiki E. (2025). Synthesis and molecular modeling of antioxidant and Anti-Inflammatory Five-Membered Heterocycle–Cinnamic acid hybrids. Molecules.

[57] Oliveira P.E.A., Silva D.P., Ferreira S.S., Marchi-Salvador D.P., Oliveira Filho A.A. (2026). Anticoagulant, antioxidant and cytotoxic potential of cinnamic acid and derivatives. Braz. J. Biol..

[58] Anantharaju P.G., Gowda P.C., Vimalambike M.G., Madhunapantula S.V. (2016). An overview on the role of dietary phenolics for the treatment of cancers. Nutr. J..

[59] Alam M.A., Subhan N., Hossain H., Hossain M., Reza H.M., Rahman M.M., Ullah M.O. (2016). Hydroxycinnamic acid derivatives: A potential class of natural compounds for the management of lipid metabolism and obesity. Nutr. Metab..

[60] Diniz L.R.L., Souza M.T.S., Barboza J.N., Almeida R.N., Sousa D.P. (2019). Antidepressant potential of cinnamic acids: Mechanisms of action and perspectives in drug development. Molecules.

[61] Ye X., Sun C., Zhao Y., Wang W., Li Z., Liu L., Han X. (2025). Promotion of sleep by cinnamic acid in parachlorophenylalanine-induced insomnia in rats. Int. Immunopharmacol..

[62] Prorok T., Jana M., Patel D., Pahan K. (2019). Cinnamic acid protects the nigrostriatal system in a mouse model of Parkinson’s disease via peroxisome proliferator-activated receptor-α. Neurochem. Res..

[63] Huang S., Liu W., Li Y., Zhang K., Zheng X., Wu H., Tang G. (2021). Design, synthesis, and activity study of cinnamic acid derivatives as potent antineuroinflammatory agents. ACS Chem. Neurosci..

[64] Tian Y., Jiang X., Guo J., Lu H., Xie J., Zhang F., Yao C., Hao E. (2025). Pharmacological potential of cinnamic acid and derivatives: A comprehensive review. Pharmaceuticals.

